# Bonesetters and Their Methods

**Published:** 1902-07-12

**Authors:** 


### BONESETTERS AND THEIR METHODS.

About thirty-five years ago the late Sir James Paget delivered a memorable lecture on "Cases that Bonesetters Cure." Now Mr. Howard Marsh, speaking at St. Bartholomew's Hospital, returns to the same subject, one which well deserves to be driven deep into the student's mind, for there can be no doubt that it is by failing to recognise that there are diseased conditions which cannot be put right without the exertion of considerable force that many surgeons not uncommonly fail to cure their patients. Mr. Howard Marsh [1] says that the cases which bonesetters cure are those in which there is not very much the matter. Serious joint-disease cannot be cured by movement. Extensive fibrous anchylosis, tuberculous disease of a joint, active osteo-arthritis, and cases in which sepsis has supervened on parturition and led to firm fibrous anchylosis, are not cases which can be cured by rough movement. The cases from which the bonesetters gain credit are those in which slight adhesions in an otherwise healthy joint have to be broken down, especially when such adhesions are outside the joint, the joint itself being normal. Mr. Marsh gives several good illustrations of the sort of cures which may be effected by the more or less rough manipulations employed by bonesetters; cures which of course ought to be effected far better by the skilled and properly directed manœuvres of medical practitioners. A gentleman undergoing treatment for a stiff shoulder at Harrogate was attacked by a cow. He attempted to escape by taking a flying leap over a hedge and ditch, and in so doing threw his arms up violently. "He not only escaped from the cow, but from that time regained free movement in his shoulder." This was probably a case of slight adhesions about the shoulder-joint ruptured by the sudden movement of the limb. The bonesetter would say, "the shoulder had gone in."

"A young gentleman strained his leg when playing tennis, and the limb remained so painful that he was unable to walk. He was sent to South Africa and back in order to rest the leg. He came back no better. Massage led to no improvement. He then went to a bonesetter and was cured immediately; for the foot was in a state of slight equinus from some tearing and subsequent adhesions of the fascia about the gastrocnemius. By his wrenching the bonesetter broke down these adhesions and cured the patient." But that is precisely the sort of case in which, had the surgeon possessed enough imagination to have been able to picture to his mind's eye the condition around the joint, the treatment required ought to have been self-evident. A gentleman fell down and hurt his elbow; synovitis followed and the arm was put on a splint and kept there, not for two or three days, but for a month. Naturally when the splint was removed the elbow was stiff. He was told, however, that it would get all right. But it did not get all right. "So the patient went to a bonesetter who said 'the bone was out,' treated it by manipulation, and the result was that in two or three days the patient was quite well." Now these are cases that we ought all to be aware of. In a case of Colles' fracture, for example, when the splints are removed the result, so far as appearances are concerned, is very good, so the doctor bids his patient good-bye. But the wrist is stiff, and at the end of three months it remains just as bad as before. "It is painful and keeps the patient awake at night, and he cannot hold a pin, or drive, or use his wrist in any way. Now is the surgeon's chance to evade the bonesetter; he should give chloroform and move the joint, and employ massage and douching."

[1] St. Bartholomew's Hospital Journal, May, 1902.

